# On the Dynamic RSS Feedbacks of Indoor Fingerprinting Databases for Localization Reliability Improvement

**DOI:** 10.3390/s16081278

**Published:** 2016-08-15

**Authors:** Xiaoyang Wen, Wenyuan Tao, Chung-Ming Own, Zhenjiang Pan

**Affiliations:** 1 School of Computer Software, Tianjin University, Tianjin 300072, China; 2014218061@tju.edu.cn (X.W.); taowenyuan@tju.edu.cn (W.T.); 2 Bohai Securities Co., Ltd., Tianjin 300072, China; panzhenjiang@hotmail.com

**Keywords:** location estimation, RSS fingerprinting, Bluetooth low energy, adaptive RSS fingerprint, feedbacks

## Abstract

Location data is one of the most widely used context data types in context-aware and ubiquitous computing applications. To support locating applications in indoor environments, numerous systems with different deployment costs and positioning accuracies have been developed over the past decade. One useful method, based on received signal strength (RSS), provides a set of signal transmission access points. However, compiling a remeasurement RSS database involves a high cost, which is impractical in dynamically changing environments, particularly in highly crowded areas. In this study, we propose a dynamic estimation resampling method for certain locations chosen from a set of remeasurement fingerprinting databases. Our proposed method adaptively applies different, newly updated and offline fingerprinting points according to the temporal and spatial strength of the location. To achieve accuracy within a simulated area, the proposed method requires approximately 3% of the feedback to attain a double correctness probability comparable to similar methods; in a real environment, our proposed method can obtain excellent 1 m accuracy errors in the positioning system.

## 1. Introduction

With the rapid development of mobile communications and pervasive computing technology, the demand for location-aware services is rapidly increasing. Although global positioning systems can provide accurate and reliable position information for location services, they cannot be used effectively indoors. Recently, numerous localization methods, such as those involving sensor networks, radio frequency identification, wireless fidelity (WiFi), Bluetooth low energy (BLE), and visual light positioning, have been proposed to overcome this limitation; among them, the use of BLE positioning technology has attracted widespread attention because it is based on mobile phones—most notably the iPhone—which are extensively used worldwide [1].

Generally, received signal strength (RSS)-based location fingerprinting adheres to the principle that each position has a unique set of signal values. When the system boots up, mobile devices receive the system’s unique RSS value; the devices subsequently search the fingerprinting database and identify the entry that is most similar to the system’s unique RSS value for the estimated location [2]. The main problem of a typical RSS fingerprinting system is that the real RSS value at any location is easily affected by the object and multipath fading effects [1]. In other words, the RSS fingerprint obtained at different times needs not match the previous fingerprint stored in the database, leading to incorrect estimation results. To overcome this problem, methods applied in utilizing fingerprinting databases should be tolerant of variance in the RSS value for a fixed position.

A technique for equalizing database fingerprinting and real fingerprints consists of remeasuring signals, called feedback, during an operating time at any location [3]. However, obtaining feedback requires a tradeoff to be achieved between the operation costs and time required. In [4], a localization system involving the use of a small amount of feedback was presented; a limitation of this system is that only feedback locations are maintained. Furthermore, Lemic et al. proposed an object comparison algorithm for the relative contributions of the individual fingerprinting phases according to the localization of their overall performances [5]; they identified a small number of WiFi access points (APs) and simple fingerprint-creation and pattern-matching procedures that could equal or outperform more sophisticated alternatives regarding localization accuracy. In save resources for achieving faster and more precise positioning, probabilistic techniques in WiFi-based systems have shown high performance. In [6], Haeberlen et al. demonstrated a system that allows for remarkably accurate localization across an entire office building over 12,000 square meters in area; most notably, regarding the scale, they localized a device to one of 510 cells in the building within seconds, yielding a success rate of greater than 95%. Moreover, Bisio et al. designed an indoor target localization scheme using electromagnetic waves; this system was replaced by an offline phase in which the fingerprints in each point of the area of interest were estimated by means of electromagnetic waves [7].

Fingerprinting is the most popular method of indoor positioning, employing a two-step procedure. The first step is the training (i.e., offline) phase, based on the collection of spatiotemporal area representations. The second is the positioning (i.e., online) phase, which starts with an online RSS measurement performed by the mobile device. In [8], the authors computed a position by considering the RSS measurements as a part of a random process, exploiting the information present in the acquired signals. In [9], Jiang et al. proposed a localization algorithm based on the concept of crucial AP. The proposed algorithm does not require an environment layout or additional equipment but computes the similarity degree on the basis of the AP distance and fingerprint repetition. Their positioning system acquired new information (i.e., feedback) from certain measured locations; the estimate was obtained using the spatial correlation between adjacent locations, an interpolation technique, and the feedback information. In this study, an adaptive RSS fingerprinting database was constructed on the basis of the following three assumptions:

(1) Locations should be close to each other should have similar RSS values.
(2) The distance between an estimated location and feedback is represented as the degree of credibility of a fingerprint obtained from the feedback.
(3) The amount of feedback in the system is the confidence ratio of the evaluation of estimated locations.

These assumptions hold for abnormal situations. Most experimental environments are defined as a spacious room without any disturbances, particularly in an extremely complex area. A suitable example is an indoor amusement park, at which people in the crowd take turns on popular rides; the obstacles in the area are moveable and changeable. Thus, a newly moving obstacle could emerge in between the instances of reference feedback. The aforementioned three assumptions are not always useful in such situations.

Generally, we refer to the data in a fingerprinting database as static, and a real-time computed value is said to be dynamic. A typical RSS location fingerprint is obtained mainly from a static database, which is ineffective in a dynamic and changeable environment. When we measure the RSS fingerprint sample at a location, the positioning algorithm might select an incorrect estimated location from the static database. The chosen entry may appear to be the most probable one for the sample; however, such an RSS value may refer to a physical location that is actually far from the real fingerprinting database.

The remainder of this manuscript is organized as follows: Section 2 reviews related studies that have investigated the properties of RSS indication (RSSI) for indoor positioning systems. Section 3 provides details of the measurement system used in the current study and describes the data analysis method. Section 4 presents the emulated and field experiments, and Section 5 offers our conclusions and recommendations for future research.

## 2. Preliminaries

### 2.1. RSS Fingerprinting

RSS properties, which facilitate location fingerprinting, have been determined by several studies on indoor positioning systems. In [10], it was observed that user orientation could cause a variation of up to 5 dBm in the RSSI level. At each location, different user and mobile device orientations with respect to the transmitter could cause the mean RSS value to differ. The modelling of RSS-based location fingerprinting is essential for location determination algorithms; examples of RSS-based location fingerprinting models are the probabilistic approach model [11] and preliminary analytical model [12]. In [9,13], Gaussian and lognormal distributions were used to model RSS randomness. For example, in a large-scale measurement, [14] explained that most RSS histograms could be fitted very well with Gaussian distribution, but that few histograms could be fitted with bimodal Gaussian distributions.

Current signal-based RSS location systems have two problems: (1) considerable manual calibration effort is required to construct a radio map in the offline training phase and (2) the positioning accuracy changes with environmental dynamics. In [15], three dynamic factors observed to change frequently over time in the environment were proposed: presence of people, relative humidity, and movement. These factors can easily affect the radio signal propagating from APs to mobile devices and are responsible for changes in positioning accuracy. 

### 2.2. Location Estimation Algorithm

In previous studies [16], methods based on the time and arrival angle of signals have not been applied because these signals are influenced by the multipath effect. However, triangulation based on attenuation methods has been used, along with models relating the received power to the propagation distance. Indoor electromagnetic wave propagation, particularly inside buildings, is defined by reflections, diffractions, and dispersion in the internal structures. The transmitted signals arrive at the receiver through multiple paths, resulting in fluctuations in the received signal. These effects, called multipath propagation, are affected by the type of materials used in the construction of buildings and surrounding objects. Hence, RSS is extremely difficult to predict.

The wall attenuation factor (WAF) model is useful for describing the slow-fading phenomenon and attenuation in signal propagation in indoor environments. In this model, the attenuation factor is used to predict the signal propagation behavior when walls are the main obstacles. The following equation shows how attenuation influences RSS:

(1) $$P{\left(d\right)}_{dbm}=P{\left(d_{o}\right)}_{dbm}-10\times n\times \log\left(\frac{d}{d_{o}}\right)-nW*WAF$$

where *n* indicates the rate of increase in the signal attenuation with propagation distance, $P\left(d_{o}\right)$ is the RSS at a distance of reference point $d_{o}$, and d is the distance between the transmitter and receiver. Furthermore, nW is the number of obstacles (i.e., walls) between the transmitter and receiver; the WAF is considered as the attenuation value resulting from the obstacles. In this equation, if nW is greater than a certain constant *C*, this constant value is considered to represent the number of walls at which the attenuation factor stops influencing the signal; we can then use the constant value instead of nW.

When we select a subset of APs satisfying a certain property in our alternative set of fingerprint definitions, we retrieve the corresponding position from the offline fingerprinting database by using the location estimation algorithm. Generally, there are several methods for determining the nearest neighbor location. For example, we can use the Euclidean distance:

$$Eucdist\left(S,R\right)=\sqrt{\sum_{i=1}^{n}{\left(s-r_{i}\right)}^{2}}$$

or the Mahalanobis distance [17]:

$$Mahaldist\left(S,R\right)=\sqrt{{\left(S-R\right)}^{T}S^{-1}\left(S-R\right)}$$

where *S* is the RSS value for the target location and *R* is the value closest to *S* in the fingerprinting database. 

### 2.3. Cutoff Area

**Figure 1:**
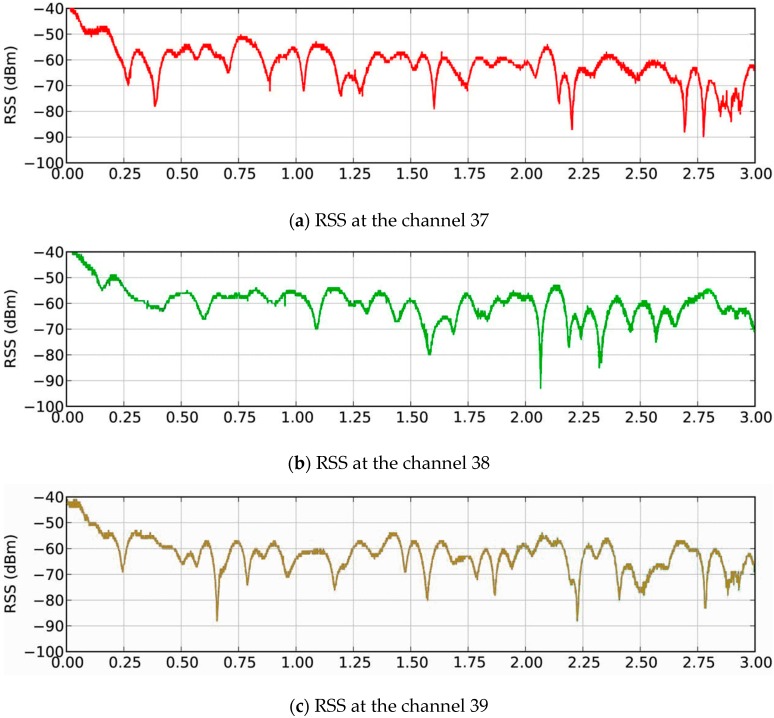
Examples of RSS variation at the channels (**a**) 37; (**b**) 38; and (**c**) 39.

In the BLE positioning system, which has 40 2-MHz-wide channels spanning the unlicensed 2.4 GHz radio band, BLE advertisements are broadcast on three highly narrow (2 MHz) advertising channels in quick succession. These three channels are nominally labelled 37, 38, and 39 and are widely spaced at 2402, 2426, and 2480 MHz, respectively (Figure 1). Figure 1 shows the RSSI values recorded by the 50 Hz BLE beacon. The mean levels of the three separated signals are different: −63.7 ± 1.9 dBm, 61.9 ± 2.3 dBm, and −67.7 ± 5.0 dBm, for channels 37, 38 and 39 respectively. Clearly, the strength of the advertising signal is not constant.

Therefore, the RSS fingerprint at a fixed location is unstable, and the RSS fingerprinting database continues to be static, rendering the database highly ineffective in a dynamic environment. When we measure an RSS fingerprint sample, the positioning algorithm might choose the most probable RSS fingerprint from the offline static RSS database. This may cause a positioning error. Generally, previous studies have used the spatial correlation of adjacent locations calculated from a set of nearby feedback locations by the linear interpolation technique [18]. For example, we have three sets from the RSS fingerprints: $p_{i}=\left(x_{i},y_{i},rss_{i}\right)$, where *i* = 1,2,3, ($x_{i},y_{i})$ are known coordinates and $rss_{i}$ is the average signal strength at the position. If we aim to predict the point $p^{*}$, which is surrounded by $p_{1},\;p_{2}\;\text{and}\;p_{3}$, we have:

$$F=\left[\begin{array}{c} x_{2}-x_{1}\\ y_{2}-y_{1}\\ rss_{2}-rss_{1} \end{array}\right]=\left[\begin{array}{c} F_{1}\\ F_{2}\\ F_{3} \end{array}\right]$$

and:

$$R=\left[\begin{array}{c} x_{3}-x_{1}\\ y_{3}-y_{1}\\ rss_{3}-rss_{1} \end{array}\right]=\left[\begin{array}{c} R_{1}\\ R_{2}\\ R_{3} \end{array}\right]$$

Accordingly, we obtain the predicted equation:

$$P^{*}=\alpha \cdot x^{*}+\beta \cdot y^{*}+\gamma \cdot rss^{*}+\delta$$

by the following predicted point $\;p^{*}$:

$$p^{*}=\left|\begin{array}{c} \begin{array}{ccc} x^{*} & y^{*} & rss^{*} \end{array}\\ \begin{array}{ccc} F_{1} & F_{2} & F_{3} \end{array}\\ \begin{array}{ccc} R_{1} & R_{2} & R_{3} \end{array} \end{array}\right|$$

where α, β, γ, and δ are arbitrary numbers, and $x^{*}$, $y^{*}$, and $rss^{*}$ are the final predicted position and strength value.

**Figure 2:**
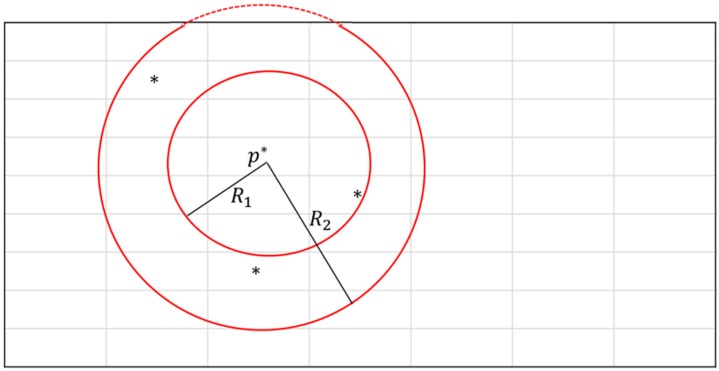
Example of cutoff areas (feedbacks are marked by *).

In [9], the authors proposed that a cutoff area can highly influence the RSS at an estimated location. The cutoff area is a circular area containing some resampling points (i.e., feedback). This circular area facilitates eliminating bias from the estimated process. Figure 2 shows the position $p^{*}$ inside the resampling area. To minimize the resampling time, the system is designed to randomly evaluate RSS values at selected positions. The radius of the cutoff area (*R*) can be adapted according to the amount of feedbacks contained. For example, in Figure 2, we have two cutoff areas enclosing point $p^{*}$, and all feedbacks are marked by an asterisk (*). We can choose the small radius $R_{1}$ for fewer feedbacks and the large radius $R_{2}$ for more feedbacks. In a given area, a greater amount of feedbacks can allow for higher precision regarding the prediction; however, the tradeoff is the system performance in generating enough feedbacks when needed.

## 3. System Design

In this study, we considered positioning environments comprising a spacious room with disturbances, particularly in an extremely complex area. A suitable example is an indoor amusement park, at which people in the crowd take turns on popular rides; the obstacles in the area are moveable and changeable. Thus, according to Assumption 1, there are exceptions for closed locations with very similar RSS values. A suitable example is a playground crowded with people who are standing side by side. Rules 1 and 2 cannot be valid in this situation. For solving this problem, we propose two tasks in the current study to manage these dynamic situations: the adaptive area estimation and dynamic feedback selection.

### 3.1. Sample Collection Phase

In our system, the estimation is based on the compilation of an adaptive RSS fingerprinting database. The estimation procedure involves referencing some feedback around $p^{*}$ in the resampling area; these procedures are repeated until all of the positions have been resampled.

**Figure 3:**
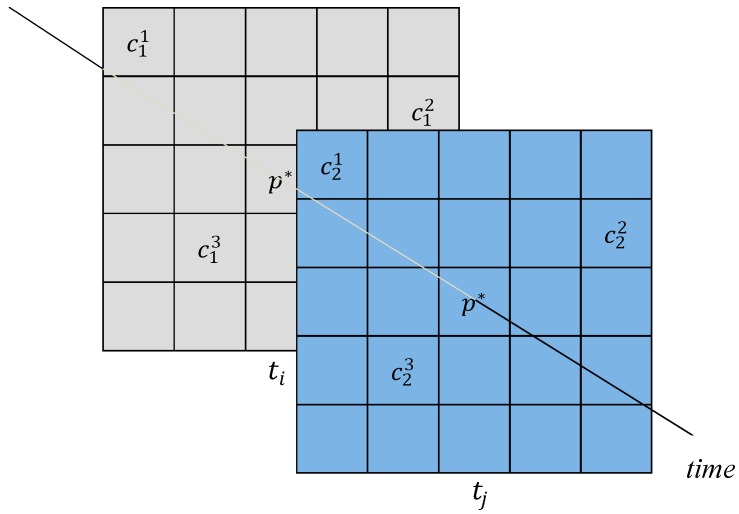
Framework of the dynamic selection.

Figure 3 shows an example of a fixed cut-off area. In some studies, such as [9], the radius of the cutoff area is fixed. However, it is apparent that the disturbance degree is positively related to the size of the cutoff area. The disturbance degree is used to represent the degree of crowding around the resampling area. A higher disturbance degree reflects greater crowding of people in the cutoff area; for correctly resampling the RSS value, more feedback is needed. However, more feedback sometimes leads to measurements with greater differences, and these results show the existence of a finite disturbance degree. Furthermore, the distance between the feedback and the APs is highly sensitive to the amount of feedback. The RSS value of a shorter distance is more likely to be correct compared with that of a larger distance.

To illustrate the aforementioned tasks, we evaluated the confidence of the feedback in the cutoff area. We can decide the extension degree of the radius according to the feedback confidence. A larger confidence value is more representative of the feedback in the cutoff area. The equation is as follows:

(2) $$confidence=\frac{10n\left(\log\left(\frac{d_{max}}{d_{0}}\right)-\log\left(\frac{d_{min}}{d_{0}}\right)\right)}{F_{max}-F_{min}}$$

$F_{min}$ and $F_{max}$ are the chosen minimum and maximum RSS values from the ranking results of the feedbacks in the cutoff area. $d_{min}$ represents the distance from the point with the minimum RSS value to the AP; $d_{max}$ denotes the distance from the point with the maximum RSS value to the AP. Furthermore, *n* is the attenuation rate of the signal, similar to the attenuation in Equation (1). In Equation (2), the numerator represents the RSS difference in the cutoff area, and the denominator denotes the difference in RSS attenuation between the path from the position with the maximum RSS to the AP and the path from the position with the minimum RSS to the AP. Because the cutoff area is the circular area containing at least three feedbacks, in our system, we first initialized the radius of the cutoff area as three units, subsequently extending the radius by applying the following the equation when the confidence value is less than 0.5:

(3) $$R_{new}=R_{O}+\lfloor \alpha \frac{1}{Confidence}\rfloor$$

where $R_{new}$ is the updated radius from the original confidence value $R_{O}$ and α is the constant (one unit in this study). The deriving procedure is repeated until there are more than two feedbacks and the corresponding confidence value is greater than 0.5.

### 3.2. Sample Calibration Phase

According to the previous assumptions, if the size of the cutoff area has been finalized, then it is fixed. The following estimation procedure is used to modify the resampling point by applying the feedback. In our assumption, we have the following rules:

(1) There is no estimation if there is no feedback.
(2) The estimated position is equal to the value of the feedback if there is a single feedback condition and it is identical to the estimated location.
(3) The value of the estimated location $p^{*}$ can be determined by using the following method if the estimated location is surrounded by feedbacks.

According to these rules, if an estimated location $p^{*}\;$ is surrounded by more than three feedback conditions (Figure 3), then dynamic selection is performed for the feedback in the proposed system. In dynamic selection, the probability of the location estimation for each feedback, obtained from the underlying localization engine, is measured.

Figure 3 shows an example of dynamic selection in our positioning system. We assume that an estimated location $p^{*}$ exists in the resampling area. We obtain the fixed radius of the cutoff area and, given three feedback conditions, our system determines the RSS values $c_{i}^{k}$ and $c_{j}^{k}$ at time instants $t_{i}$ and $t_{j}$, respectively; here, *k* = 1, 2, and 3 and $t_{i}<t_{j}$. The factors $\alpha_{k}=1/d_{k}$ and $\beta_{k}=c_{j}^{k}/c_{i}^{k}$ are defined and employed to represent two relationships of feedback *k*, and $d_{k}\;$is the distance from the feedback *k* to the AP; this value can be computed by Equation (1).

(1) Spatial relationship: If $\beta_{k}\;$is large, it implies that feedback *k* has a large RSS value from $t_{j}$ to $t_{i}$, that the disturbances around feedback *k* were recently reduced, and that feedback *k* is more reliable than the other feedback conditions. If $\beta_{k}\;$ is small, the reverse holds true.
(2) Temporal relationship: If $\alpha_{k}$ is large, this indicates that feedback *k* has a large RSS value and that the feedback is reliable in the cutoff area. If $\alpha_{k}$ is small, the reverse is true.

We express the dynamic selection equation of *k* in the cutoff area as follows:

(4) $$DS_{k}=\frac{\alpha_{k}\beta_{k}}{\sum_{r=1..n}\alpha_{r}\beta_{r}}$$

where *n* denotes the amount of feedback in the cutoff area. Finally, the RSS value of the *estimated location* $p^{*}$ is defined as:

(5) $$RSS_{p^{*}}=\sum_{r=1..3}DS_{r}\cdot c_{j}^{r}$$

Thus, according to the Equation (5), we can modify the resampled RSS value in a limited operation.

## 4. Experiments and Discussion

The following two experiments were performed to evaluate the proposed adaptive location system. In the first experiment, we attempted to simulate the environment of an indoor amusement park where people take turns on popular rides; we also introduced obstacles to the area. Because these obstacles were changeable, we applied the simulation tool MATLAB to simulate an obstacle-free space with an area of 30 × 30 m, divided into 900 locations, and an AP at each corner. The minimum distance between two adjacent locations was 1 m. In the second experiment, we built our test environment in Building 55 in Tianjin University (Tianjin, China). The space had an area of 5 × 15 m, divided into 30 locations, and four APs. Each individual location had an area of 1.6 × 1.6 m. In the first experiment, we tested the fingerprints of all 900 locations to evaluate the system, and, in the second experiment, 30 locations were considered and employed as a RSS fingerprinting database for the training phase. In the positioning phase, each location generated a new RSS fingerprint, which was used in a search algorithm to calculate the estimated location and determine the percentage of correctly estimated locations. In all experiments, the proposed method was compared using a plane interpolation method (proposed in [9], herein referred to as the interpolation method) and a randomly modified sampling method [13].

### 4.1. Simulated Experiment

In the simulation, we designed a space with an area of 30 × 30 m (900 locations) to test the proposed system. The size of this space was too large to allow for constant resampling the fingerprinting database. Moreover, we aimed to simulate moving obstacles (i.e., people in the crowd) in an indoor amusement park; therefore, we applied Equation (1) with different WAF values to consider different levels of crowding. The value WAF = 0 implied that there was almost no crowd in the amusement park, and the values WAF = 1.1, 3.2, and 6.4 respectively represented light, heavy, and very heavy crowds; furthermore, each WAF value respectively corresponded to crowds typically observed in the morning, afternoon, evening, and midnight hours on holidays at amusement parks.

**Figure 4:**
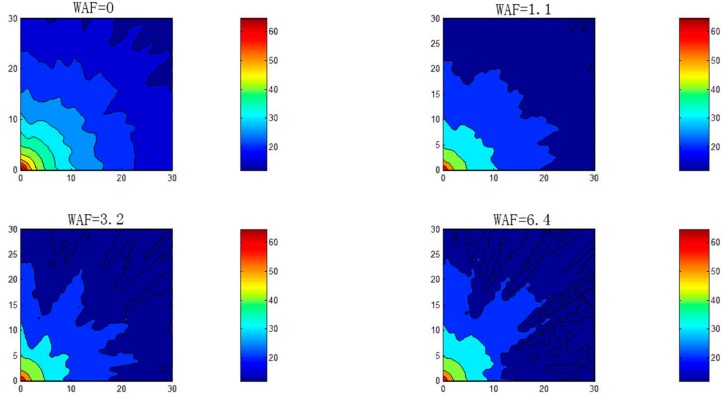
Crowd effects with one AP.

**Figure 5:**
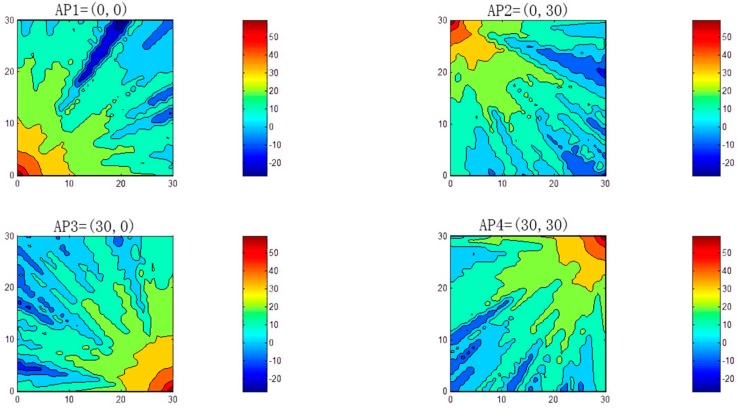
WAF relationship among APs; AP1 is in the upper left, AP2 is in the upper right, AP3 is in the lower left, and AP4 is in the lower right.

Figure 4 indicates that the crowds appeared randomly in the test area. The APs were positioned at the four corners of the space. To simulate a real environment, at the beginning of the experiment, we randomly selected some obstacle placements. For example, in Figure 4a, the panel for WAF = 0 represents an irregular fan-shaped map of the signal strength at the left-bottom corner of the AP, indicating that fixed obstacles actually blocked the signal in the environment. Figure 4b–d represent a situation where the wireless signal was blocked randomly by crowds, excluding the fixed obstacles in the environment. The simulation showed that signal mappings were difficult to model or trace in the presence of obstacles in the environment, readable fan-shaped map of the environment was retained. Figure 5 indicates a signal strength mapping for heavy crowds of WAF = 6.4 in the simulated environment; the four panels represent the four corners where the APs were positioned. In this figure, we can deduce the limits of the system modeling in the RSS detecting environment; some details are similar, whereas others are not.

**Figure 6:**
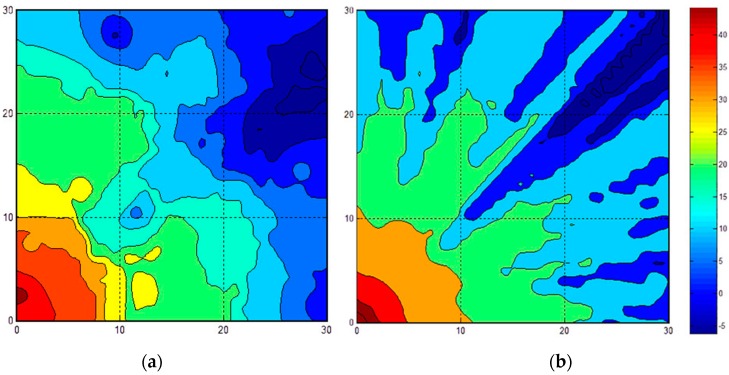
WAF = 6.4 with 30 feedbacks. (**a**) The strength mapping using our proposed method (**b**) The total resampling result with Equation (1).

**Figure 7:**
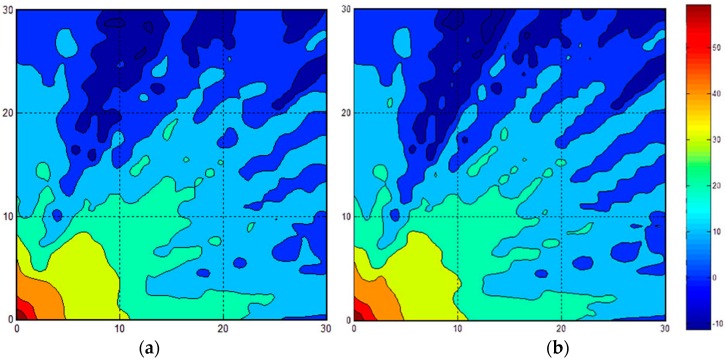
WAF = 6.4 with 600 feedbacks. (**a**) The strength mapping using our proposed method (**b**) The total resampling result with Equation (1).

Because the simulation environment was too large to resample all of the RSS fingerprints, we employed the proposed system in this area. In other words, we retrieved parts of the feedback to modify the online fingerprinting database. We simulated the environment with light and very heavy crowd characteristics of an indoor amusement park; in other words, we applied WAF = 1.1 and 6.4 to Equation (1). In the simulated area, some obstacles were fixed in the map. Subsequently, we randomly selected 30 points to establish the feedback shown in Figure 6. Figure 6 exhibits a comparison of 30 feedback conditions in the proposed method when complete resampling and heavy crowds were applied. Figure 6a is the strength mapping with 30-feedback remeasuring using our proposed method, whereas Figure 6b is the total resampling result with Equation (1). The resampling operation time could save 29/30, and the fan-shaped cycle is still retained in Figure 6a. Furthermore, Figure 7 shows a similar comparison of 600 feedback conditions. Figure 7a represents a mapping with 600 feedbacks, whereas Figure 7b presents the total resampling result. The saving time was 1/3. For the situation considered, the signal strength map should show the same fan-shaped cycle for the optimal results. The fan-shaped cycle in Figure 6a is not as regular as that in Figure 6b because the resampling size is limited to 30 points. However, the fan-shaped cycle in Figure 7a is similar to that in Figure 7b, indicating that the system can save one third of the operation time under conditions with greater amounts of feedback (e.g., 600-feedback conditions).

**Figure 8:**
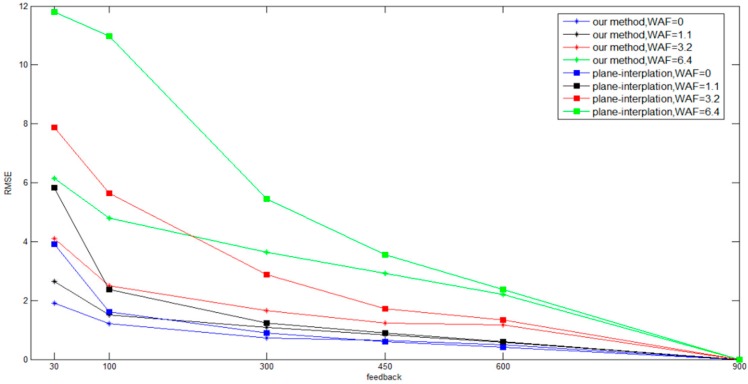
Comparison results with different methods.

Figure 8 compares the obstacle blocking results of our proposed method and interpolation method for different amounts of feedback. According to the RMSE values, the proposed method not only saves system time but also considerably increases the positioning accuracy when there is less feedback. Furthermore, a higher amount of online resampling feedbacks entails a greater degree of similarity between the accuracies of the proposed method and the interpolation method because a higher amount of online resampling feedback increases the positioning accuracy and there is no dynamic feedback to select.

### 4.2. Field Experiment

**Figure 9:**
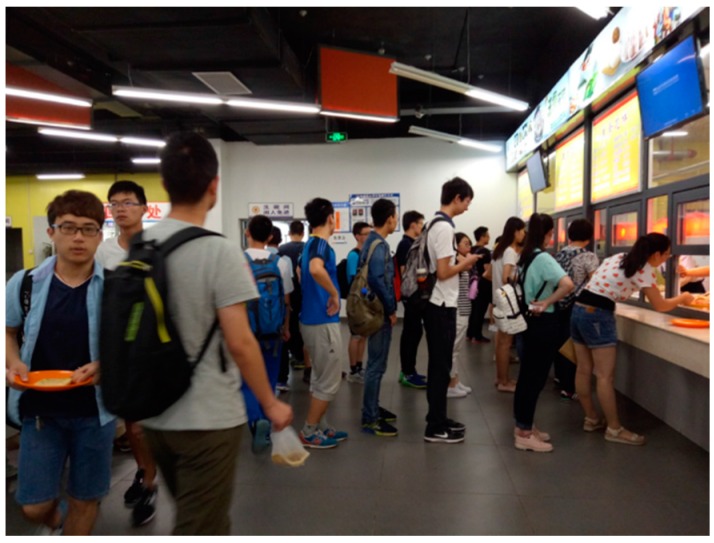
Students lined up in the no. 3 student cafeteria of Tianjin University.

To demonstrate the proposed method, we created a test environment at the No. 3 cafeteria of Tianjin University (Tianjin, China). The space considered had an area of 3.6 × 20 m (divided into 42 blocks) and six APs. Each block had an area of 1.2 × 1.4 m. We positioned the APs at the four corners and two middle pillars of the space. This area was the food service location, there were food-serving stalls, either in a line or allowing arbitrary walking paths. To test our system idea, our field experiments were conducted from 11:00 to 13:20 in this area, representing the time at which the space is most crowded, students are eager for food, Figure 9 was the real view in the cafeteria. Initially, we collected all of the RSS fingerprinting databases for each AP in advanced. It took us about 2.5 h each time for the total investigation. 

**Figure 10:**
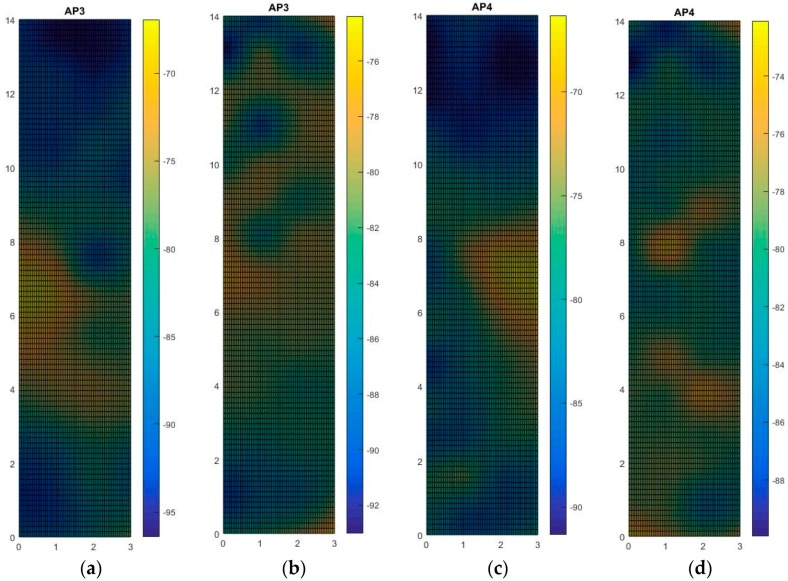
The RSS strength mapping of AP3 and AP4, (**a**) no students with AP3; (**b**) crowded students with AP3; (**c**) no students with AP4; (**d**) crowded students with AP4.

**Figure 11:**
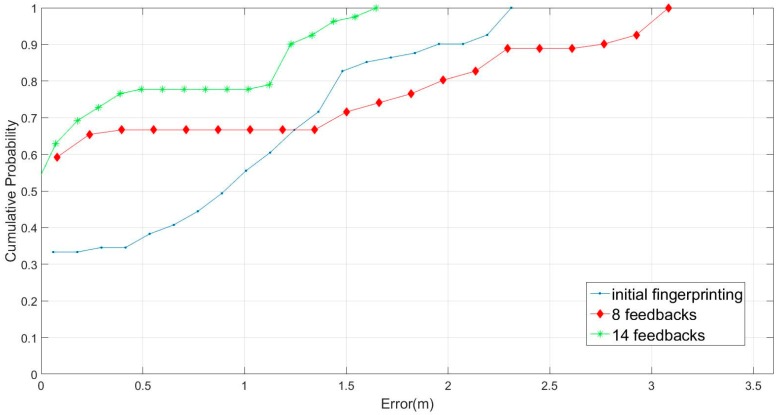
Field experiment with eight- and fourteen-feedbacks.

**Figure 12:**
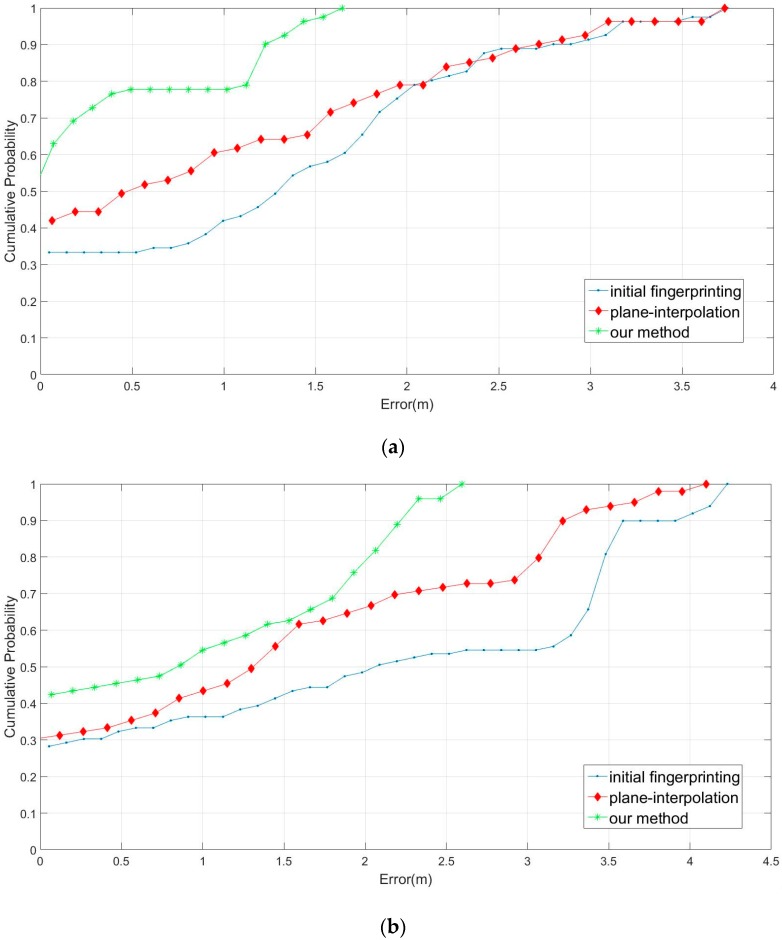
Field experiment (**a**) without and (**b**) with obstacles in the cafeteria.

Accordingly, we executed our remeasuring experiment in the lunch time, students were crowded and lined up in front of the stalls, these people were the obstacles in our experiments. The RSS strength mapping is presented in Figure 10, to save the reading spaces, we only demonstrate the mapping with AP3 and AP4. The results show that the crowded students really changed the signal strength mapping. Firstly, we used the aforementioned fingerprinting database and adopted six- and nine-feedback conditions in a remeasuring experiment. The comparisons of online accuracy were based on 11 positions chosen randomly. The experiment computed the online prediction based on the three methods, including original fingerprinting values, referring by eight- and fourteen- feedbacks. The results (Figure 11) demonstrated that our system could reduce operating time and increase positioning accuracy with the average error 1.48 m, 1.2 m and 0.4 m respectively. Furthermore, we compared our proposed method with the interpolation method in the testing environment, with and without obstacles, as shown in Figure 12.

Figure 12a presents the comparison results without obstacles in the initial fingerprinting database, employing our proposed method and the interpolation method with eight feedbacks (no students in the cafeteria). Moreover, Figure 12b presents the comparison results with obstacles. The performance of our method was superior to that of the interpolation method (crowded students in the cafeteria). The results in Figure 12a, evidence that our proposed method exhibited a superior performance; the sampling errors were almost all located within 1.5 m. By contrast, the plane interpolation method had an approximately 63% sampling error rate within 1.5 m; the initial fingerprinting database method had an approximately 58% error rate within 1.5 m, the performance was a little better than guessing. In Figure 12b, the sampling error rates within 1 m were 63%, 58%, and 43% for our proposed method, plane interpolation method, and initial fingerprinting database, respectively. Besides, our predicting results were no worse than 2.3 m, however, the other two methods were worse than the error in 4 m. The results showed that the adaptive radius was the main factor influencing location positioning, indicating that our proposed method demonstrated optimal performance in fingerprinting database rebuilding. The average errors are listed in Table 1. All of the comparisons were evaluated for the 10-time database remeasuring. The results showed that our method can enhance performance in the resampling fingerprinting database.

## 5. Conclusions

Generally, the main challenge in RSS-based location positioning is the high sensitivity of this technique to environmental changes. Variations in the RSS measurement hinder the estimation accuracy. In other words, if the radio propagation signal strength was correlated for the distance between the transmitter and receiver, location determination would be a minor problem. However, the relationship between these two parameters is dynamic rather than straightforward. In this study, we proposed a new algorithm for adapting the RSS fingerprinting database by using surrounding feedback information, besides, our system employs different, newly updated remeasuring and offline fingerprinting points according to the temporal and spatial strengths of the locations. Because our system applies the adaptive radius as the main factor influencing location positioning, the signal blocking from moving obstacles can be solved in the simulated and real testing environments. Accordingly, for achieving an accuracy within 30 × 30 m and a location resolution of 1 m in a simulated area, the proposed method requires approximately 3% of the feedback to attain a double correctness probability comparable to similar methods; furthermore in the 3.6 × 20 m real testing environment in the crowded cafeteria, our proposed method could retain the excellent 1.5 m accuracy error in the positioning system.

## Figures and Tables

**Table 1:** Average positioning error at the no. 3 cafeteria of Tianjin University.

	Initial Fingerprinting Database	Eight-Feedbacks		Fourteen-Feedbacks	
		Plane-Interpolation Method	Our Proposed Method	Plane-Interpolation Method	Our Proposed Method
Without Obstacles	1.48 m	1.35 m	1.2 m	0.92 m	0.4 m
With Obstacles	2.38 m	2.15 m	1.98 m	1.58 m	1.16 m
